# Quantification of US Food and Drug Administration Premarket Approval Statements for High-Risk Medical Devices With Pediatric Age Indications

**DOI:** 10.1001/jamanetworkopen.2021.12562

**Published:** 2021-06-22

**Authors:** Samuel J. Lee, Lauren Cho, Eyal Klang, James Wall, Stefano Rensi, Benjamin S. Glicksberg

**Affiliations:** 1 Hasso Plattner Institute for Digital Health at Mount Sinai, Icahn School of Medicine at Mount Sinai, New York, New York; 2 Institute for Healthcare Delivery Science, Icahn School of Medicine at Mount Sinai, New York, New York; 3 Department of Surgery, Stanford University, Stanford, California; 4 School of Medicine, Stanford University, Stanford, California; 5 Department of Genetics and Genomic Sciences, Icahn School of Medicine at Mount Sinai, New York, New York

## Abstract

**Importance:**

Medical device companies submit premarket approval (PMA) statements to the US Food and Drug Administration (FDA) for approval of the highest-risk class of devices. Devices indicated for the pediatric population that use the PMA pathway have not been well characterized or analyzed.

**Objective:**

To identify and characterize high-risk devices with pediatric age indications derived from PMA statements.

**Design, Setting, and Participants:**

In this cross-sectional study of PMA statements, those statements containing the words *indicated* or *intended* for medical devices listed in the FDA PMA database as of February 2020 were retrieved. Age indications were manually annotated in these approval statements via PubAnnotation. Based on the PMA identification from the PMA statements, device metadata including product codes, regulation numbers, advisory panels, and approval dates were queried.

**Main Outcomes and Measures:**

The main outcome was discernment of the distribution of devices indicated for the pediatric population (neonate, infant, child, and adolescent). Secondary measures included outlining the clinical specialties, device types, and lag time between the initial approval date and the first date of an approval statement with a pediatric indication for generic device categories.

**Results:**

A total of 297 documents for 149 unique devices were analyzed. Based on the manual age annotations, 102 devices with a pediatric indication, 10 with a neonate age indication, 32 with an infant age indication, 60 with a child age indication, and 94 with an adolescent age indication were identified. For indications for patients from age 17 to 18 years, the number of devices available nearly doubled from 42 devices to 81 devices. Although more than half of the surveyed devices had a pediatric age indication, many were available only for a limited range of the pediatric population (age 18-21 years). For indications for patients from age 0 to 17 years, the mean (SD) number of clinical specialties at each age was 7.27 (1.4), and 12 clinical specialties were represented from ages 18 to 21 years.

**Conclusions and Relevance:**

In this cross-sectional study on device PMA statements, a gap was identified in both quantity and diversity of high-risk devices indicated for the pediatric population. Because the current scarcity of pediatric devices may limit therapeutic possibilities for children, this study represents a step toward quantifying this scarcity and identifying clinical specialties with the greatest need for pediatric device innovation and may help inform future device development efforts.

## Key Points

**Question:**

Can a database compiled from premarket approval statements for high-risk medical devices be used to characterize pediatric age indications as well as device types and specialties represented?

**Findings:**

In this cross-sectional study including 297 premarket approval (PMA) documents for 149 unique devices, 102 devices (68%) contained a pediatric age indication that predominantly consisted of adolescent age (12 years to 21 years) indications.

**Meaning:**

In this cross-sectional study quantifying and characterizing PMA documents, a gap was found in availability of pediatric medical devices, suggesting that most devices were developed for the adolescent pediatric population.

## Teaser

This cross-sectional study identifies and characterizes high-risk devices with pediatric age indications derived from US Food and Drug Administration (FDA) premarket approval statements.

## Introduction

Medical devices in the US are regulated by the US Center for Devices and Radiological Health of the US Food and Drug Administration (FDA) for quality, safety, and effectiveness.^1^ The FDA categorizes medical devices into 3 classes (I, II, and III) in order of risk. Class III designation is reserved for devices that “support or sustain human life, are of substantial importance in preventing impairment of human health, or that present a potential, unreasonable risk of illness or injury.”^2^ These high-risk devices often require premarket approval (PMA), which has stringent testing requirements to demonstrate safety and effectiveness.^3,4^ In contrast, a 510(k) premarket submission is used for medical devices that are “substantially equivalent” to previously approved devices.^5^ These devices tend to be class II and have a precedent for safety and effectiveness. Class I devices are usually exempt from review.

Substantial barriers exist in developing medical devices for the pediatric population. These include a lack of pediatric device trials infrastructure, difficulty in enrolling pediatric participants, and high costs.^6^ An FDA-led national survey of government-associated clinicians found that 74% of device needs pertain to the pediatric population.^7^ The report further found that clinicians with a pediatric focus were more likely than those without one to modify or repurpose a therapeutic device or use the device for off-label treatment in patients. Off-label use has emerged as a by-product of the relative scarcity of specifically approved pediatric medical devices and the device needs of the pediatric population.^8^ The extent of off-label use in pediatric populations and the effectiveness and safety of off-label use are not well characterized.

The integration of medical devices in clinical settings requires attention to their indications and use. Off-label use in pediatric clinical care is relatively common because few devices are specifically indicated for use in pediatric clinical care populations across clinical disciplines.^9^ Off-label device use is problematic, especially in the context of high-risk class III devices. For example, biliary stents and embolization coils are regularly used off-label in pediatric interventional cardiology and can lead to severe clinical complications, such as intravascular hemolysis, embolization, and thrombosis.^10^ The pediatric population has unique medical needs with relevant differences in growth and development that preclude the direct application of adult devices.^11^

A systematic quantitative analysis of age-based device availability may help highlight areas of need for innovators and policy makers, characterize rates and off-label use in pediatric patients, and facilitate the implementation and assessment of targeted policies and initiatives to address disparities. However, this type of analysis has not been conducted to date because no database has comprehensively compiled the age-based indications for medical devices. This information is dispersed across regulatory documents, such as the FDA reports to Congress and medical-device labels in free-text descriptions of indications for use, making systematic analysis a challenge.^12^

The aim of this study was to quantify and characterize high-risk medical devices with pediatric age indications. We hypothesized that fewer medical devices would be available for the pediatric population. We compiled a database of age indication information from PMA statements and used it to characterize the number and types of high-risk devices that are intended for use in pediatric patients.

## Methods

### Data Set Retrieval and Preprocessing

We retrieved PMA statements that included the words *indicated* or *intended* for medical devices listed in the FDA PMA database from inception to February 2020 using the OpenFDA REST API.^13^ We also obtained metadata associated with PMA supplements, including product codes, regulation numbers, advisory panels, and approval dates. We then preprocessed the text of approval order statements by natural language processing systems by removing characters not found in the American Standard Code for Information Interchange, such as trademark symbols, converting text written in all capital letters to sentence case, and correcting segmentation errors for enumerated lists. We released the corpus of approval order statements on PubAnnotation.^14^ This study was exempt from institutional review board approval according to the general policies of the Icahn School of Medicine at Mount Sinai institutional review board regarding research not involving human participants.

**Figure 1.**
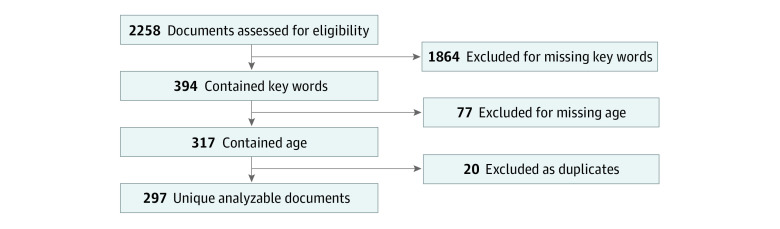
Flow Diagram of Premarket Approval Statements With Age-Related Information

After initial data cleaning of the PMA statements, we searched for key words pertaining to age, including *age*, *pediatric*, *adolescent*, *neonate*, *infant*, *child*, *children*, *younger*, *older*, and *years*, for a total of 394 documents (Figure 1). Duplicates and documents without age indication were removed, with a final sample of 297 viable statements for analysis. This study followed the Strengthening the Reporting of Observational Studies in Epidemiology (STROBE) reporting guideline for cross-sectional studies.

### Annotation Guidelines

We developed guidelines to standardize the manual annotation process using an iterative approach. Starting from a simple initial system, we annotated all text spans pertaining to age-based populations, identified constituent semantic types and linguistic features of age indications, and updated our annotation guidelines until we reached a final set of guidelines. Updating the guidelines was done collaboratively among 4 researchers (S.J.L., L.C., S.R., and B.S.G.), and changes were accepted only in the case of unanimous agreement. The final set of semantic types used for annotation included age category, age range, age range start, age range start unit, age range end, and age range end unit. The annotation guidelines are available in the eMethods in the Supplement.

### Manual Annotation

We manually annotated text spans denoting age indications according to our annotation guidelines using TextAE.^15^ To ensure the validity of our natural language processing system, 2 reviewers (S.J.L. and L.C.) independently annotated each PMA statement document. Where we observed discrepancies between reviewers, a third individual (B.S.G) resolved the difference to create a final consensus annotation set that was used in our final analysis.

### Statistical Analysis

After filtering the initial corpus to 297 unique documents, we downloaded and further cleaned the annotations using Python programming language version 3 (Python Software Foundation). Annotations with the same meaning but different spellings were standardized across documents for each annotation category. For instance, the phrases “18 years of age and older” and “18 years and older” were standardized to “18 years of age and older.” Age annotations that were spelled out were converted to their numeric equivalents (ie, “eighteen” was converted to “18”). Age ranges in devices indicating multiple age ranges were collapsed into an age range that included all specified age ranges.

Under section 515A of the Federal Food, Drug, and Cosmetic Act, the FDA classifies the pediatric population as birth through 21 years of age and further subdivides this population into neonate, infant, child, and adolescent.^16^ The FDA categorizes neonates from birth through the first 28 days, infants from 29 days to 2 years of age, children from 2 years to 12 years of age, and adolescents from 12 years to 21 years. Because each subpopulation presents with its own unique challenges,^17^ we classified devices as indicated for pediatric patients or adult patients and further classified devices indicated for pediatric patients into pediatric subgroups. Some devices were classified into multiple groups because of age indications that intersected with multiple predefined FDA age groups. We then tabulated the number of devices available for patients at each age from 0 to 21 years. Using the device metadata on the advisory committee, we then stratified devices by clinical specialty to investigate which clinical specialties were represented.^18^

We calculated the time between the initial PMA statement and the PMA statement with a pediatric indication for various generic device categories. We identified the generic device category from the metadata for each identified pediatric device based on the manual annotation process.^19^ We queried the OpenFDA API for the date of the first PMA statement for that generic category. We then manually surveyed the subsequent PMA statements for a given generic device category to find the date of approval for the first mention of a pediatric indication. Using these 2 dates, we calculated the difference between the 2 dates for each pediatric generic device category.

## Results

Of the 149 unique devices analyzed, we identified 102 devices (68%) with a pediatric indication, 10 devices (7%) with a neonate age indication, 32 devices (21%) with an infant age indication, 60 devices (40%) with a child age indication, and 94 devices (63%) with an adolescent age indication.

### Manual Review Process

An example of our annotations in PubAnnotation is shown in the eMethods in the Supplement, identifying key words related to age according to our annotation guidelines (eFigure 1 in the Supplement).

This consensus set for the 297 documents consists of a total of 1568 annotations distributed across the 5 annotation groups agreed on in the annotation guidelines. The most common annotations were age end (349 of 1568 [22%]), age start (346 of 1568 [22%]), and age range (333 of 1568 [21%]). Fewer documents had unit (age start unit: 235 of 1568 [15%]; age end unit: 58 of 1568 [4%]) and age category (247 of 1568 [16%]) to annotate (eFigure 2 in the Supplement).

The device statements surveyed contain a wide scope of age ranges indicated for multiple age groups. The mean (SD) number of devices for the pediatric population aged 21 and younger was 47.13 (19.63) devices. However, many of these devices were indicated for patients aged 18 and over. The number of devices with an age indication from 17 years to 18 years increased from 42 to 81 (Table 1). The increases in devices may owe to the fact that the most common age ranges used in the approval statements were 18 years of age and older and 21 years of age and older.

**Table 1.** Number of Devices With Age Indication for Ages 0 to 21 Years or Older by Clinical Specialty

Clinical specialty	Devices, No. (%)^a^																						
	0 (n=22)	1 (n=27)	2 (n=35)	3 (n=35)	4 (n=38)	5 (n=38)	6 (n=39)	7 (n=42)	8 (n=44)	9 (n=41)	10 (n=41)	11 (n=41)	12 (n=42)	13 (n=40)	14 (n=41)	15 (n=41)	16 (n=42)	17 (n=42)	18 (n=81)	19 (n=81)	20 (n=81)	21 (n=103)	>21 (n=138)
Ophthalmology	0	0	1 (2)	1 (2)	1 (3)	1 (3)	1 (3)	1 (2)	2 (4)	2 (5)	2 (5)	2 (5)	2 (5)	1 (3)	1 (2)	1 (2)	1 (2)	1 (2)	10 (12)	10 (12)	10 (12)	18 (17)	36 (26)
Cardiology	7 (32)	9 (33)	10 (29)	10 (29)	12 (31)	12 (31)	12 (31)	12 (28)	13 (30)	10 (24)	10 (24)	10 (24)	10 (24)	9 (23)	9 (22)	9 (22)	9 (21)	9 (21)	18 (22)	18 (22)	18 (22)	18 (17)	18 (13)
Immunology	1 (4)	1 (4)	1 (2)	1 (2)	1 (3)	1 (3)	1 (3)	1 (2)	1 (2)	1 (2)	1 (2)	1 (2)	1 (2)	1 (3)	1 (2)	1 (2)	1 (2)	1 (2)	1 (1)	1 (1)	1 (1)	1 (1)	17 (12)
Orthopedics	0	0	0	0	0	0	0	0	0	0	0	0	0	0	0	0	0	0	1 (1)	1 (1)	1 (1)	1 (1)	2 (1)
Ear, nose, throat	1 (4)	5 (19)	6 (17)	6 (17)	6 (16)	6 (16)	6 (16)	6 (14)	6 (13)	6 (15)	6 (15)	6 (15)	7 (17)	7 (18)	7 (17)	7 (17)	7 (17)	7 (17)	12 (15)	12 (15)	12 (15)	12 (12)	12 (9)
Clinical chemistry	1 (4)	0	3 (8)	3 (8)	3 (8)	3 (8)	4 (10)	7 (16)	7 (16)	7 (17)	7 (17)	7 (17)	7 (17)	7 (18)	8 (20)	8 (20)	9 (21)	9 (21)	15 (18)	15 (18)	15 (18)	15 (15)	15 (11)
Microbiology	9 (41)	10 (37)	11 (31)	11 (30)	11 (30)	11 (30)	11 (28)	11 (26)	11 (25)	11 (27)	11 (27)	11 (27)	11 (26)	11 (28)	11 (27)	11 (27)	11 (16)	11 (16)	11 (14)	11 (14)	11 (14)	12 (12)	9 (7)
Surgery	0	0	0	0	0	0	0	0	0	0	0	0	0	0	0	0	0	0	3 (4)	3 (4)	3 (4)	12 (12)	12 (9)
Neurology	0	0	0	0	1 (3)	1 (3)	1 (3)	1 (2)	1 (2)	1 (2)	1 (2)	1 (2)	1 (2)	2 (5)	2 (5)	2 (5)	2 (5)	2 (5)	6 (7)	6 (7)	6 (7)	9 (9)	9 (7)
Radiology	0	0	0	0	0	0	0	0	0	0	0	0	0	0	0	0	0	0	0	0	0	0	1 (1)
Anesthesiology	1 (4)	0	0	0	0	0	0	0	0	0	0	0	0	0	0	0	0	0	0	0	0	0	0
Gastroenterology, urology	0	0	1 (2)	1 (2)	1 (3)	1 (3)	1 (3)	1 (2)	1 (2)	1 (2)	1 (2)	1 (2)	1 (2)	0	0	0	0	0	1 (1)	1 (1)	1 (1)	2 (2)	2 (2)
Obstetrics/gynecology	2 (9)	2 (7)	2 (5)	2 (5)	2 (5)	2 (5)	2 (5)	2 (5)	2 (4)	2 (5)	2 (5)	2 (5)	2 (5)	2 (5)	2 (5)	2 (5)	2 (5)	2 (5)	2 (2)	2 (2)	2 (2)	2 (2)	2 (2)
Pathology	0	0	0	0	0	0	0	0	0	0	0	0	0	0	0	0	0	0	0	0	0	0	2 (2)
Physical medicine	0	0	0	0	0	0	0	0	0	0	0	0	0	0	0	0	0	0	1 (1)	1 (1)	1 (1)	1 (1)	1 (1)

^a^ All percentage values were rounded to whole numbers.

### Device Landscape

The 297 documents reviewed accounted for 149 unique devices with PMA statements because some documents were supplements updating the information on a device model. Of these devices, 102 devices (68%) had a pediatric age indication (under 21 years), 10 devices (7%) had a neonate age indication (birth to 28 days), 32 devices (21%) had an infant age indication (29 days to 2 years), 60 devices (40%) had a child age indication (2-12 years), and 94 devices (63%) had an adolescent age indication (12-21 years). Because devices can be indicated for broad age ranges, some devices are indicated for multiple pediatric subgroups; 140 of the 149 identified devices (94%) were class III, and 9 devices (6%) were class II.

### Results by Clinical Specialty

Fifteen different clinical specialties were represented by the device statements reviewed (eTable 1 in the Supplement). Of these specialties, the most frequently represented were ophthalmology with 48 devices, cardiology with 22, immunology with 16, and clinical chemistry with 16, whereas radiology, anesthesiology, and physical medicine had 1 associated device each. After stratification for clinical specialty, we found that the diversity of clinical specialties with age-approved devices increased with increasing age (Table 1). From ages 0 to 17 years, the mean (SD) number of specialties represented was 7.27 (1.4) of the total 15 clinical specialties. In contrast, 12 of 15 specialties were represented from ages 18 to 21 years. From age 17 to 18 years, notable increases in ophthalmology (from 1 to 10), neurology (from 2 to 6), and clinical chemistry (from 9 to 15) were seen. In addition, surgery, orthopedics, radiology, pathology, and physical medicine devices had no representation from ages 0 to 17. Adults aged 21 years and older had the greatest representation across specialties, with 14 (93%) of 15 specialties represented in devices available.

### Pediatric Subpopulation Analysis

We classified devices into pediatric subgroups based on identified age indications; 40 of the 94 identified adolescent devices (43%) were not indicated for children, infant, or neonate age groups. Few devices were approved specifically for the children (27), infant (17), and neonate (10) age ranges (eTable 2 in the Supplement). This finding is consistent with the higher number of devices available to patients aged 18 years and over.

The clinical specialties most associated with devices with a pediatric indication were cardiology (22), ophthalmology (18), and clinical chemistry (16) (Table 2). The most common devices seen with a pediatric indication include an excimer laser system (13), hepatitis B screening tests (11), automated external defibrillators (7), invasive glucose sensors (7), and cochlear implants (5) (Table 3). A total of 100 of the pediatric devices (98%) were class III and the remaining 2 (2%) were class II.

**Table 2.** Number of Devices With Pediatric Age Indication by Clinical Specialty

Clinical specialty	No. of devices
Cardiology	22
Ophthalmology	18
Clinical chemistry	16
Microbiology	12
Ear, nose, throat	12
General, plastic surgery	8
Neurology	6
Gastroenterology, urology	2
Obstetrics/gynecology	2
Physical medicine	1
Anesthesiology	1
Immunology	1
Orthopedics	1

**Table 3.** Number of Pediatric Generic Device by Category

Device class	No. of devices
Excimer laser system	13
Hepatitis B screening test	11
Invasive glucose sensor	7
Automated external defibrillator (nonwearable)	7
Cochlear implant	5
Dermal implant for aesthetic use	5
Shock-wave generator for pain relief	4
Percutaneous catheter cardiac ablation for atrial flutter	4
Percutaneous catheter cardiac ablation	3
Automated insulin dosing, threshold suspend	2
Fetal pulse oximeter	2
Transcatheter septal occluder	2
Insulin infusion pump used with invasive glucose sensor	2
Middle ear hearing implant, partially implanted	2
High-frequency ventilator	1

#### Neonate

For the 10 identified devices with a neonatal age indication, all were hepatitis B screening tests that belonged to the clinical specialties of microbiology or immunology. All were class III devices.

#### Infant

For the 32 identified devices with an infant age indication, the most common clinical specialties were cardiology and otolaryngology. Most cardiovascular devices were automated external defibrillators, and most otolaryngology devices were cochlear implants. Thirty-one of these devices (97%) were class III, and 1 device (3%) was class II.

#### Children

Of the 60 devices, the most common clinical specialties were cardiology and clinical chemistry. Most of the cardiovascular devices were automated external defibrillators, and most of the clinical chemistry devices were insulin pumps and glucose sensors. A total of 58 of the devices (97%) identified were class III, and 2 devices (3%) were class II.

#### Adolescent

Of the 94 devices identified for adolescents, the most common clinical specialties were cardiology and clinical chemistry. Most of the cardiovascular devices were automated external defibrillators, and most of the clinical chemistry devices were insulin pumps and glucose sensors. Excimer laser system devices also made up the most common device accounting for increased ophthalmic devices represented among the adolescent devices. Ninety-two of the devices (98%) were class III and 2 devices (2%) were class II.

### Indication Time Lag Results by Device Category

**Figure 2.**
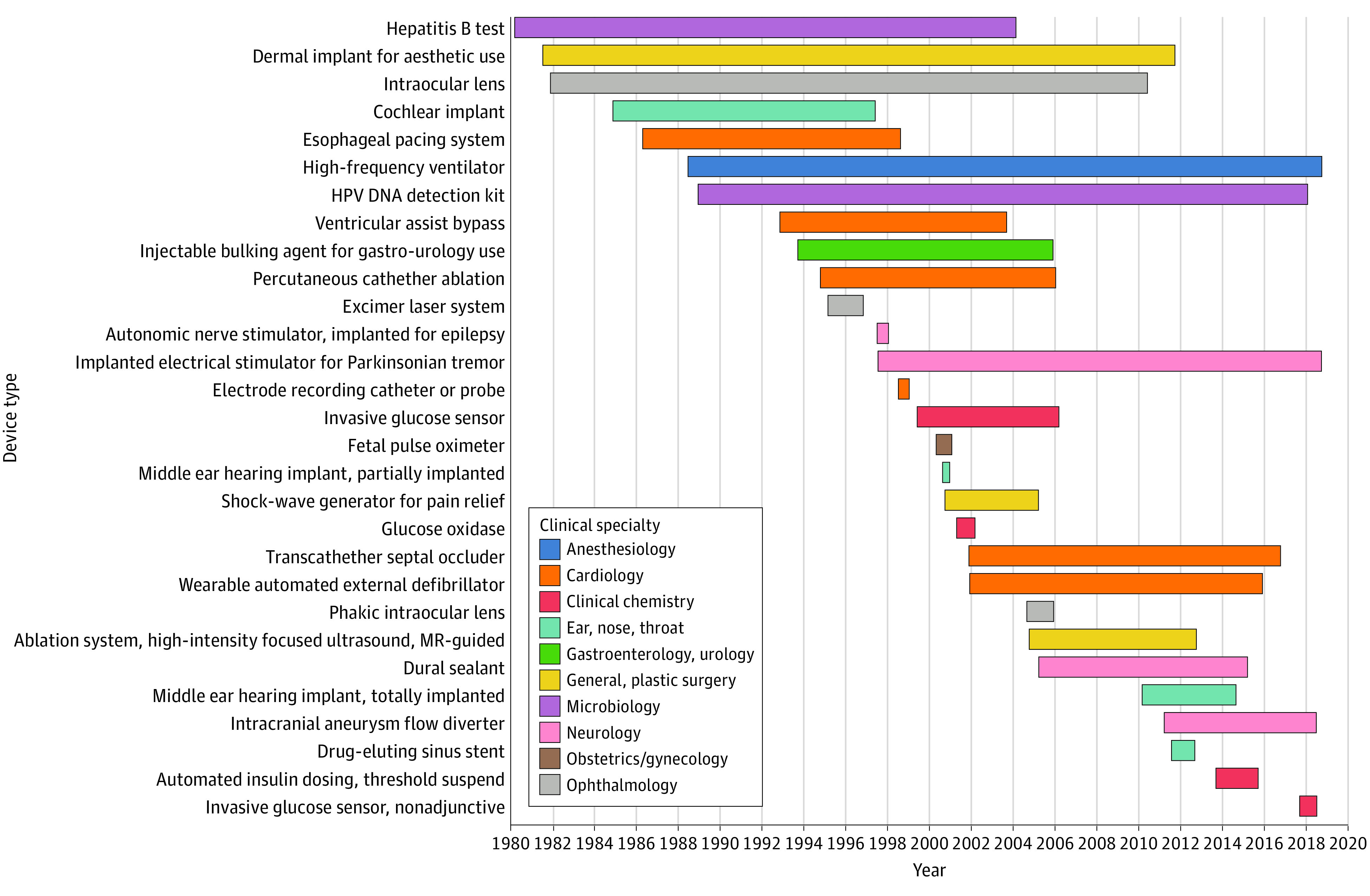
Lag Time Between First-in-Class PMA Statement and First PMA Statement With Pediatric Indication HPV DNA indicates human papillomavirus DNA; MR, magnetic resonance; PMA, premarket approval.

By examining the time between a generic device category’s initial approval and its first approval with a pediatric indication, we quantified the lag time among different device categories. A total of 18 (38%) generic device categories were approved for a portion of the pediatric population during their initial approval, and for others, years passed before a pediatric indication was included in its approval statement. The wide heterogeneity in device approval times highlights the difficulties in regulating and identifying gaps in pediatric device innovation (Figure 2).

## Discussion

In this cross-sectional study of pediatric indications for PMA statements for medical devices, we characterized the gap in both quantity and diversity of devices approved for use in the pediatric population vs the adult population. Only 42 devices with a PMA statement were identified for use in pediatric patients under the age of 18 years. A notable increase was seen at the ages of 18 and 21, showing a wide gap in the number of devices for the younger pediatric population compared with adults. This gap is particularly problematic given the vulnerable population and the life-sustaining nature of these devices. Devices approved for older adolescents are generally devices approved for adults, which does not address the unique needs of the younger pediatric subgroups. Our study is consistent with the finding that most devices indicated for the pediatric population are limited to those over 18 years of age.^20^

Distinction between the pediatric subpopulations is important because the safety and efficacy profile of a device may vary across pediatric subpopulations from neonates to young adolescents.^21^ Devices indicated for use in adolescents had the greatest range of clinical specialties represented, while devices indicated for use in children, infants, and neonates had a less diverse range of specialties represented. For instance, we found no devices indicated for ages 0 to 17 years in surgery, orthopedics, radiology, pathology, and physical medicine. This disparity was consistent with the high proportion of pediatric devices that are indicated for the age group of 18 to 21 years. Notably, our analysis identifies clinical specialties that are most in need of pediatric device innovation.

With the increasing role of medical devices in modern medicine and the diverse and unique needs of the pediatric population, pediatric device development is important to the field of pediatrics. A number of policies have been implemented to address the need for pediatric devices. Pediatric device consortiums throughout the US provide funding and in-kind support to innovators developing new pediatric devices.^6,22^ Targeted nondilutive funding through the Small Business Innovation Research and Small Business Technology Transfer programs may also provide powerful incentives for developers to prioritize pediatric populations.^23,24^ The System of Hospitals for Innovation in Pediatrics–Medical Devices initiative attempts to provide a comprehensive strategic plan to address the regulatory, financing, hospital, and reimbursement challenges associated with medical device development.^25^ In addition to these policies, the use of real-world data are another important measure to address the scarcity of evidence in pediatric devices.^26^ Real-world data, including electronic health records, claims, and data from mobile devices, can be used to support expanded indications for preapproved devices or postmarketing surveillance of devices in use.^27,28^

We noted variability in descriptions of age ranges and age-based populations in the PMA statements, which made analysis of this information challenging. Although the FDA issued draft guidance^29^ and a proposed rule for manufacturers submitting a PMA statement,^30^ further standardization or enforcement may be warranted. Standardization of age indications of devices may help reduce uncertainty about the indicated subpopulations for a device. Increased clarity in age indication descriptions in PMA statements along with improved accessibility and centralization of data for age indication is required to optimally evaluate the pediatric medical device landscape.

### Limitations

Our study has limitations. We used the FDA-defined guidelines for the pediatric subgroups, but these guidelines may not subdivide the pediatric population in a developmentally or physiologically meaningful manner. Another limitation is that we did not account for devices approved for use in pediatric patients when age was not mentioned in PMA statements. In addition, we analyzed PMA devices, which make up only 10% of available devices.^32^ We believe we have laid the groundwork for a much larger analysis of class II and humanitarian device exemption devices.

## Conclusions

To our knowledge, this is the most comprehensive characterization of the pediatric high-risk device landscape. Given the segmented nature of information on pediatric devices, we hope this analysis and annotation database may serve as a resource to characterize high-risk pediatric devices. Future studies may annotate PMA statements for other device indication information, such as anatomy, disease, procedure, molecular entities, and contraindications. In addition, we released the natural language processing annotation guidelines, the annotated data set corpus, and all code to promote open science. We have highlighted several areas of need for pediatric device innovation.

## Notes

Supplement. **eMethods.** Annotation Guidelines **eFigure 1.** Annotation Example Using PubAnnotation **eFigure 2.** Countplot of Annotation Categories **eTable 1.** Number of Clinical Specialties Associated **eTable 2.** Number of Devices Available by Pediatric Subgroup
