# Supplementary material for: Quantification of US Food and Drug Administration Premarket Approval Statements for High-Risk Medical Devices With Pediatric Age Indications
Source: JAMA Netw Open. 2021 Jun 22;4(6):e2112562. doi: 10.1001/jamanetworkopen.2021.12562 (PMC8220494; doi:10.1001/jamanetworkopen.2021.12562)
Supplement: Supplement. — eMethods. Annotation Guidelines eFigure 1. Annotation Example Using PubAnnotation eFigure 2. Countplot of Annotation Categories eTable 1. Number of Clinical Specialties Associated eTable 2. Number of Devices Available by Pediatric Subgroup [file jamanetwopen-e2112562-s001.pdf]

Supplemental Online Content

Lee SJ, Cho L, Klang E, Wall J, Rensi S, Glicksberg BS. Quantification of US Food and Drug Administration premarket approval statements for high-risk medical devices with pediatric age indications. *JAMA Netw Open*. 2021;4(6):e2112562. doi:10.1001/jamanetworkopen.2021.12562

eMethods. Annotation Guidelines
eFigure 1. Annotation Example using PubAnnotation
eFigure 2. Countplot of Annotation Categories
eTable 1. Number of Clinical Specialties Associated
eTable 2. Number of Devices Available by Pediatric Subgroup

This supplemental material has been provided by the authors to give readers additional information about their work.

© 2021 Lee SJ et al. *JAMA Network Open*.

eMethods. Annotation Guidelines

Below are guidelines in annotating age for FDA Approval statements for devices.

Age_range: A range of values that includes a lower and upper bound. The upper or lower bound does not have to be a specific number.
Ex: 22 years or older
In this example, the whole phrase would be annotated as age_range

Age_start: The lower bound of an age range.
Ex: 12-months through 17 years, 11-months of age.
In this example, the phrase, “12” would be annotated as age_start

Age_start_unit: The unit of the lower bound of the age range
Ex: 12-months through 17 years, 11-months of age.
In this example, the phrase, “months” would be annotated as age_start_unit

Age_end: The upper bound of an age range.
Ex: 12-months through 17 years, 11-months of age.
In this example, the phrase, “17” and “11” would be annotated as age_end as two separate annotations.
Words like “older”, “greater than”, “up to” indicating some upper bound should be annotated as “age_end.” Therefore age_end does not have to be a number.

Note: The full phrase “12-months through 17 years, 11-months” would be annotated as age_range.

Age_end_unit: The unit of the upper bound of the age range.
Ex: 12-months through 17 years, 11-months of age.
In this example, the phrase, “years” and “months” would be annotated as age_end_unit as two separate annotations.

Age_category: Key words about a specific age population that the device is indicated for which can include: pediatric, adult, adolescent, infant, child/children, neonate
*** Do not annotate these subpopulations: male, female, pregnant, and more.

© 2021 Lee SJ et al. *JAMA Network Open*.

A

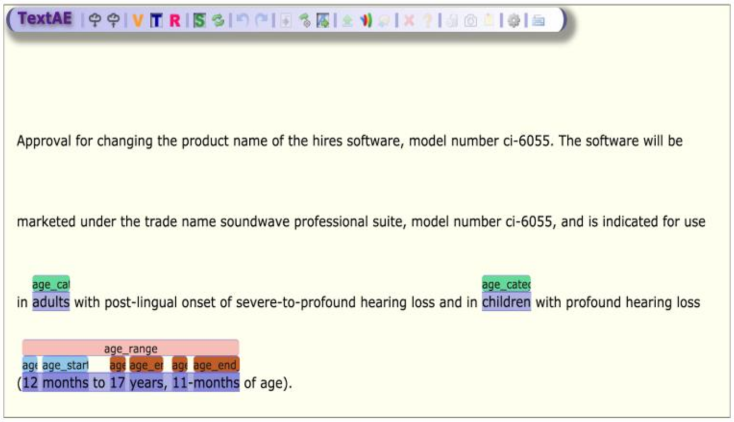

B

Annotated Word/Phrase	Annotated Category
adults	age_category
children	age_category
12	age_start
months	age_start_unit
17	age_end
years	age_end_unit
11	age_end
months	age_end_unit
12 months to 17 years, 11-months	age_range

e Figure 1. Annotation Example using PubAnnotation

A) An example of the PubAnnotation interface is shown with example annotations based on annotation guidelines. B) The annotated word or phrase along with the associated annotated category is reflected in this table. The reviewers independently highlight relevant keywords and assign them to the predefined categories based on the annotation guidelines. As seen, certain words can be part of multiple annotations.

© 2021 Lee SJ et al. *JAMA Network Open*.

Manual Annotation

From the consensus set of annotations we found that the most common annotation categories were age_end, age_start, and age_range.

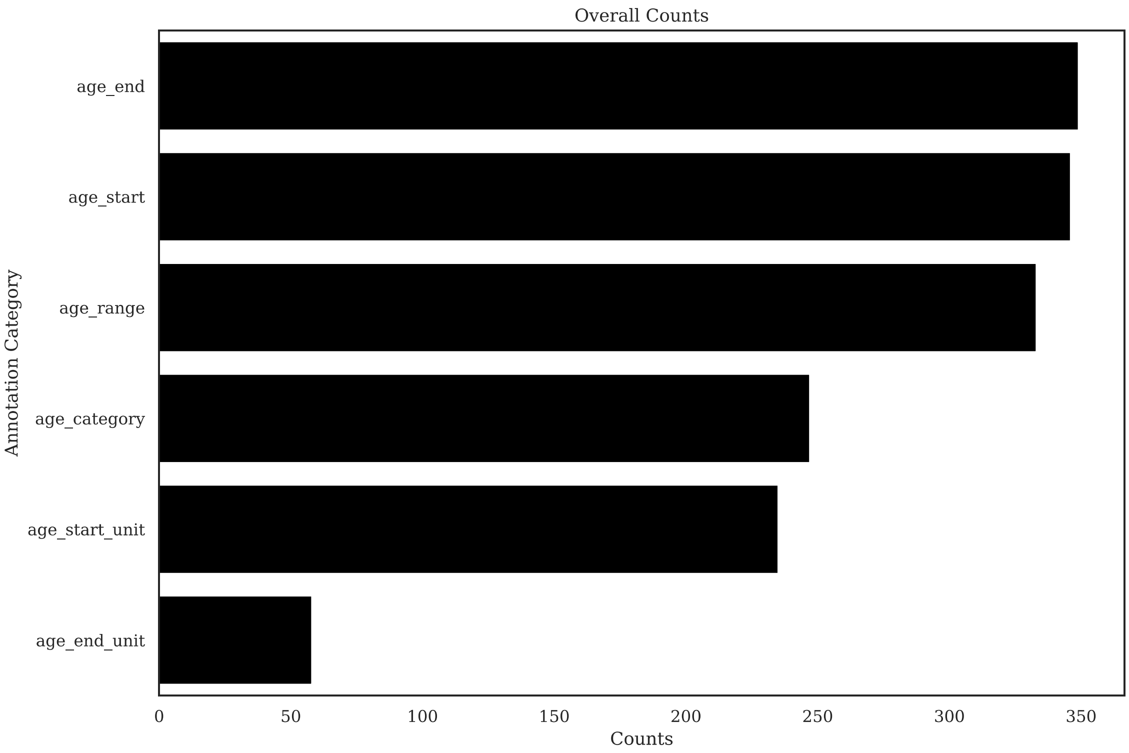

eFigure 2. Countplot of Annotation Categories

The distribution of the number of annotations shows a predominance of age start, age end, and age range annotations in the surveyed documents. Many documents had multiple age ranges to be annotated leading to a larger number of annotations than documents. Documents also had multiple forms of expressing the same age annotation. For example, a document containing “4 (four) years of age.” would have the number, “4” and the word, “four” annotated as the start of the age range.

© 2021 Lee SJ et al. *JAMA Network Open*.

We have listed below the specific fields used in our analysis from the device metadata from the openFDA API. Based on the PMA, we linked this device metadata with our age annotated dataset.

- Applicant
- Street Address
- City
- State
- Zip Code
- Generic Name
- Trade Name
- Product Code
- Advisory committee
- Supplement type
- Supplement reason
- Expedited review flag
- Date Received
- Decision Date
- Docket Number
- Federal regulation notice date
- Decision code
- Approval Statement
- Device Name
- Device Class
- Regulation Number

© 2021 Lee SJ et al. *JAMA Network Open*.

We used the advisory committee field from the device metadata to categorize devices by clinical specialties. Below represents the overall counts of each clinical specialty for each unique PMA represented.

**Clinical specialty**	**Number of Devices**
Ophthalmic	48
Cardiovascular	22
Immunology	16
Clinical Chemistry	16
Ear, Nose, Throat	12
General, Plastic Surgery	12
Neurology	9
Microbiology	4
Gastroenterology, Urology	3
Obstetrics/Gynecology	2
Orthopedic	2
Pathology	2
Radiology	1
Physical Medicine	1
Anesthesiology	1

eTable 1. Number of Clinical Specialties Associated

© 2021 Lee SJ et al. *JAMA Network Open*.

	Neonates and Older	Infants and Older	Children and Older	Adolescents Only
Number of Devices	10	17	27	40

eTable 2. Number of Devices available by Pediatric Subgroup

© 2021 Lee SJ et al. *JAMA Network Open*.
